# The significance of membrane fluidity of feeder cell-derived substrates for maintenance of iPS cell stemness

**DOI:** 10.1038/srep11386

**Published:** 2015-06-12

**Authors:** Yue Zhou, Hongli Mao, Binata Joddar, Nobuhisa Umeki, Yasushi Sako, Ken-Ichi Wada, Chieko Nishioka, Eiki Takahashi, Yi Wang, Yoshihiro Ito

**Affiliations:** 1 Nano Medical Engineering Laboratory, RIKEN, 2-1 Hirosawa, Wako, Saitama 351-0198, Japan; 2 School of Nursing, Nanjing University of Chinese Medicine, 138 Xianlin Road, Qixia District, Nanjing, Jiangsu Province 210023, China; 3 Department of Regenerative Medicine, School of Pharmaceutical Science, Jilin University, No.1266 Fujin Road, Changchun 130021, China; 4 Cellular Informatics Laboratory, RIKEN, 2-1 Hirosawa, Wako, Saitama 351-0198, Japan; 5 Support Unit for Animal Experiment, Research Resources Center, RIKEN Brain Science Institute, 2-1 Hirosawa, Wako, Saitama 351-0198, Japan; 6 Emergent Bioengineering Materials Research Team, RIKEN Center for Emergent Matter Science, 2-1Hirosawa, Wako, Saitama 351-0198, Japan

## Abstract

The biological activity of cell-derived substrates to maintain undifferentiated murine-induced pluripotent stem (iPS) cells was correlated to membrane fluidity as a new parameter of cell culture substrates. Murine embryonic fibroblasts (MEFs) were employed as feeder cells and their membrane fluidity was tuned by chemical fixation using formaldehyde (FA). Membrane fluidity was evaluated by real-time single-molecule observations of green fluorescent protein-labeled epidermal growth factor receptors on chemically fixed MEFs. Biological activity was monitored by colony formation of iPS cells. Treatment with a low concentration of FA sustained the membrane fluidity and biological activity, which were comparable to those of mitomycin C-treated MEFs. The biological activity was further confirmed by sustained expression of alkaline phosphatase, SSEA-1, and other pluripotency markers in iPS cells after 3–5 days of culture on FA-fixed MEFs. Chemical fixation of feeder cells has several advantages such as providing ready-to-use culture substrates without contamination by proliferating feeder cells. Therefore, our results provide an important basis for the development of chemically fixed culture substrates for pluripotent stem cell culture as an alternative to conventional treatment by mitomycin C or x-ray irradiation.

The surrounding microenvironments of stem cells have been investigated in detail and the factors affecting stem cell fate are also a focus of stem cell biology^1^,^2^,^3^,^4^,^5^. Such factors are investigated in terms of biological and physical aspects using biological and synthetic substrates. From a biological viewpoint, many extracellular matrices (ECMs) and adhesion proteins have been investigated in such studies. However, recent studies have revealed the significance of the static properties of substrates, such as their mechanical and topological effects on stem cell fate^6^,^7^,^8^,^9^,^10^,^11^,^12^,^13^,^14^,^15^. However, the preparation of an artificial niche has mainly focused on ECMs or their derivatives^16^,^17^, although a natural niche is formed by the cells and ECM.

Information exchanged between cells is generally in the form of chemical signals or proteinous interactions that occur through various receptors and ligands in the cell membrane^18^. Receptor-ligand interactions at the cell surface are the first steps in cellular signaling. Because receptor activation and deactivation are initiated in the plasma membrane, the membranous environment likely affects receptor activation, signal propagation, and the processes involved in receptor deactivation. Membrane fluidity influences these processes either through global effects on the physical state of the membrane matrix, such as micro-viscosity changes, or specific chemical interactions of boundary lipids with receptor proteins and transmitters^19^. A recent study showed that a difference in the mobility of membrane ligands affects both the clustering and activities of the corresponding receptor molecules^20^.

The relationship between membrane fluidity and protein activation has been investigated extensively^21^,^22^,^23^,^24^. Recent reports have shown the significance of molecular mobility in cell adhesion and morphology^25^,^26^,^27^,^28^. Such dynamic behavior is considered to be important for static properties of materials, such as elasticity and rigidity. However, the significance of this dynamic behavior has not been investigated in stem cell culture.

In pluripotent stem cell (PSC) culture, the undifferentiated state is generally maintained *in vitro* by co-culture with appropriate non-proliferative feeder cells that mimic the PSC niche, which are prepared by irradiation or mitomycin C (MMC) treatment of mouse embryonic fibroblasts (MEFs)^29^,^30^,^31^,^32^. However, these feeder cells have two practical problems. First, the preparation procedure is laborious. Live feeder cells must be prepared for every passage. Second, feeder cells contaminate the culture during passaging and harvesting of PSCs. To overcome these problems, a storable ready-to-use culture substrate has been developed using formaldehyde (FA)- or glutaraldehyde (GA)-fixed MEFs. Embryonic stem (ES) cells and induced pluripotent stem (iPS) cells grow well on chemically fixed MEFs while maintaining their undifferentiated state^33^,^34^.

Previous studies have also shown that membrane proteins retain their biological activity after chemical fixation^35^,^36^,^37^,^38^,^39^. Furthermore, direct cell–cell interactions among PSCs and feeder cells play an important role in the maintenance of PSCs. Because proteins can be retained on the plasma membrane of feeder cells after fixation, fixed feeder cells can be considered as nurse cells that support the undifferentiated growth of stem cells. However, it is still unclear why feeder cells retain their biological activities after chemical fixation.

Considering direct interactions of PSCs with fixed feeder cells occur at the cell surface, the physical properties of the cell membrane are likely related to their biological activities. However, to the best of our knowledge, no studies have investigated the relationship of the activities of chemically fixed feeder cells and the physical properties of the cell membrane. Recently, Tanaka *et al*.^40^ reported that cell membrane components, such as transmembrane proteins, lipid-anchored proteins, and lipids, maintain their mobility after FA or GA fixation. This finding suggests that the sustained functionality of chemically fixed MEFs is because of the preservation of molecular movement or membrane fluidity after fixation. In this study, we investigated the relationship of the mobility of the cell membrane and the biological activities of FA-fixed MEFs as nurse cells for mouse iPS (miPS) cell culture.

## Results

### Membrane fluidity of FA-fixed MEFs

**Figure 1:**
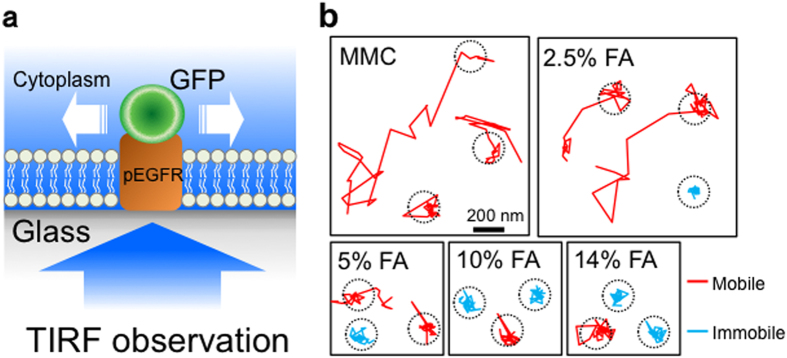
Single-molecule measurement of a membrane protein in FA-fixed MEFs. (**a**) Schematic of the method for single-molecule measurement. EGFR-GFP molecules expressed in MEFs were observed at a single-molecule resolution by TIRF microscopy after MMC treatment or FA fixation. (**b**) Examples of single-molecule movements of EGFR-GFP. The molecular movement was traced at a temporal resolution of 30.5 msec for 6 s (200 frames). In this measurement, molecules that expanded in the >200-nm range and stayed in the <200-nm range were regarded as mobile and immobile molecules, respectively. Dotted circles represent the 200-nm range.

**Figure 2:**
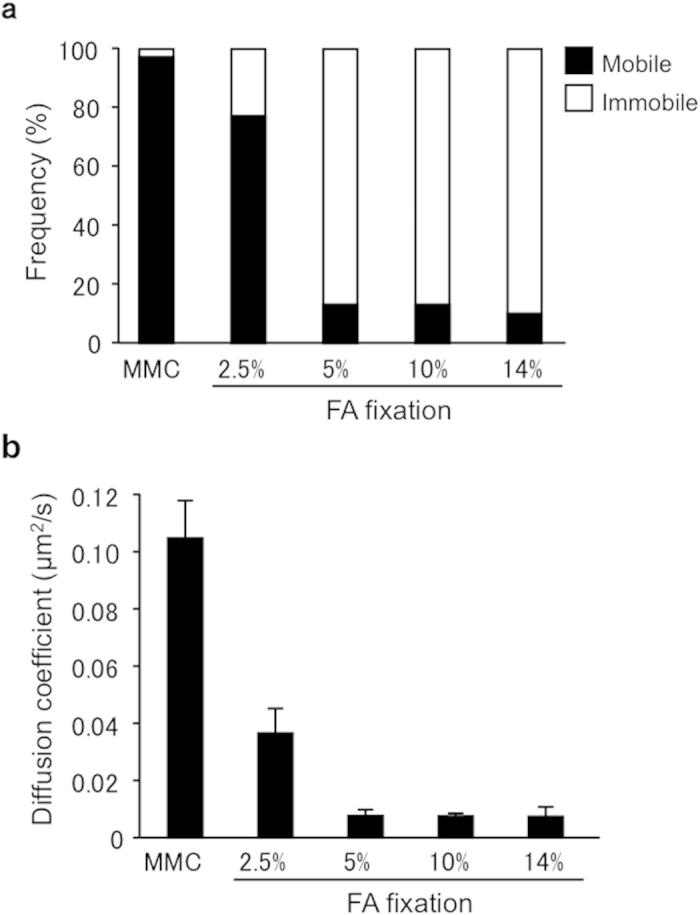
Membrane fluidity of FA-fixed MEFs. (**a**) The frequency of mobile (solid) and immobile (outline) EGFR-GFP molecules in MMC-treated and 2.5–14% FA-fixed MEFs. (**b**) The diffusion coefficient was calculated from the EGFR-GFP molecular movement data (see Materials and Methods). Values are the means ± SD, n = 3.

Total internal reflection fluorescence (TIRF) microscopy enables us to monitor the movement and spatiotemporal localization of fluorescently labeled single molecules in living cells^41^,^42^,^43^,^44^. In this study, we observed the movement of single EGFR-GFP molecules on the plasma membrane using TIRF microscopy. We first evaluated the frequency of mobile and immobile molecules (Fig. 1). The frequency of mobile EGFR-GFP in 2.5% FA-fixed MEFs was similar to that in MMC-treated MEFs. Conversely, ≥5% FA treatment significantly reduced the frequency of mobile EGFR-GFP molecules (Fig. 2a). The membrane diffusion coefficient was calculated from the EGFR-GFP movement. In MMC-treated MEFs, EGFR-GFP molecules diffused rapidly on the cell membrane (*D* = 0.105 ± 0.012 μm^2^/s), indicating the diffusive property of a live cell membrane. The 2.5% FA-fixed MEFs also maintained the diffusive property of their cell membrane (*D* = 0.037 ± 0.009 μm^2^/s), whereas ≥5% FA fixation resulted in almost complete elimination of the diffusive property (*D* < 0.008 μm^2^/s) (Fig. 2b).

Considering both the frequency of mobile and immobile molecules and the diffusive property, the membrane fluidity of 2.5% FA-fixed MEFs was more similar to that of non-fixed cells compared with 5% FA-fixed cells. A previous study reported that membrane molecules can move even after fixation by 4% paraformaldehyde^40^. We confirmed the molecular movement in 2.5% FA-fixed MEFs using EGFR-GFP molecules as a representative cell membrane protein.

### Effect of the FA concentration on MEF properties

To examine whether membrane fluidity is related to the functionality of feeder cells, we compared the capacities of 2.5–14% FA-fixed MEFs with MMC-treated MEFs to support undifferentiated growth of miPS cells.

**Figure 3:**
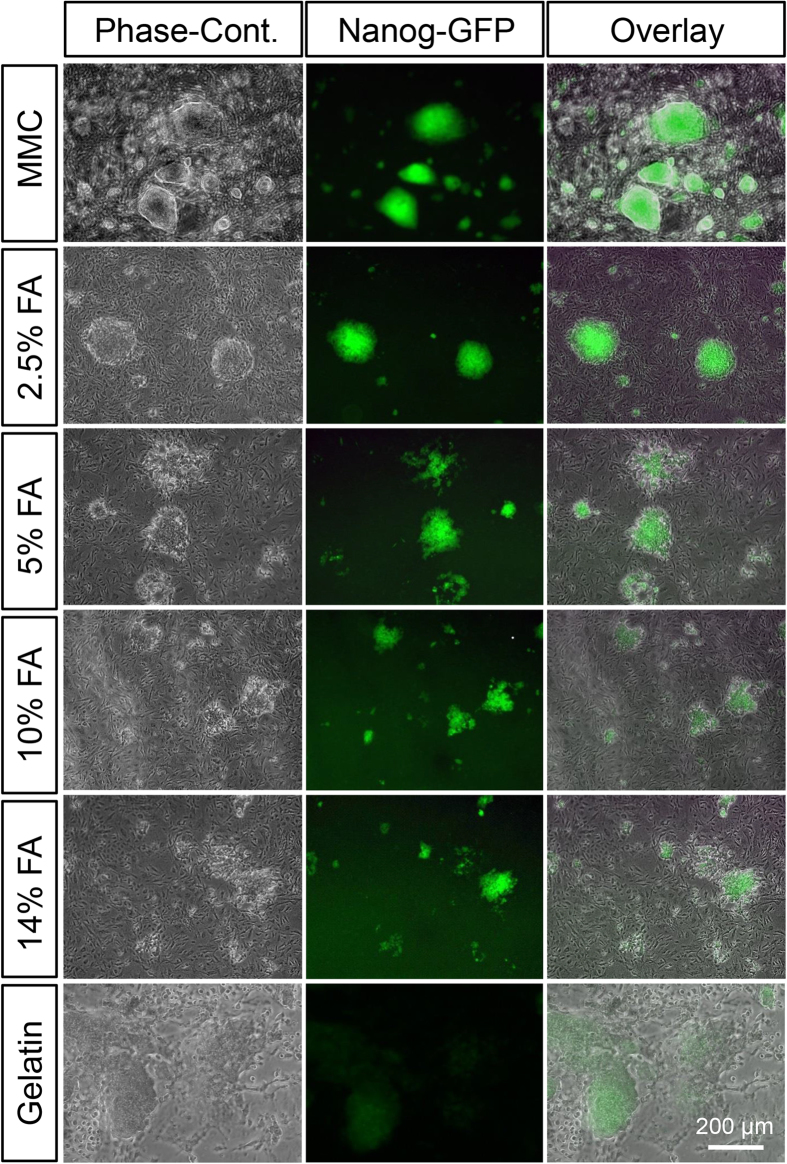
Morphology and Nanog-GFP expression of miPS cells cultured on MMC-treated MEFs, 2.5–14% FA-fixed MEFs, or gelatin-coated surfaces. Green indicates Nanog-GFP expression. MMC: MEFs treated with 10 μg/mL MMC for 3 h; 2.5–14% FA: MEFs fixed with 2.5–14% FA for 10 min; Gelatin: miPS cells cultured on gelatin-coated dishes without feeder cells.

Figure 3 shows that the colony morphology and Nanog expression level of miPS cells cultured on MMC-treated or 2.5–14% FA-fixed MEFs and gelatin-coated dishes. Nanog expression was observed in miPS cells that were cultured on MMC-treated or 2.5–4% FA-fixed MEFs. However, differentiated colonies were frequently observed on ≥5% FA-fixed MEFs and gelatin-coated dishes.

**Figure 4:**
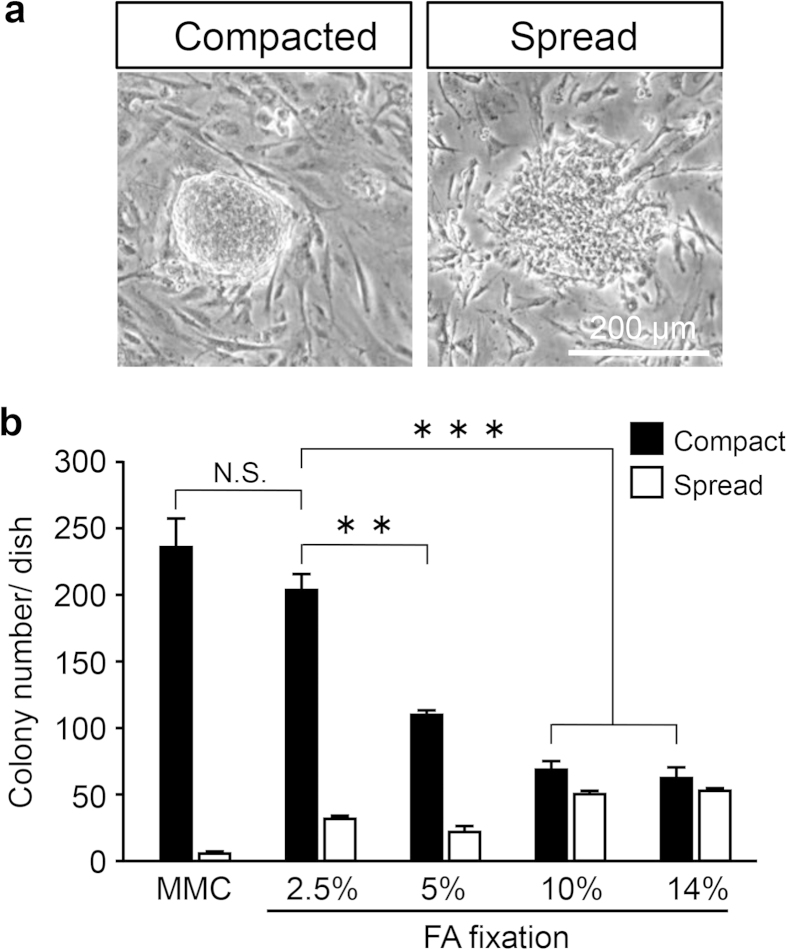
Colony formation assay of chemically fixed MEFs. (**a**) Typical examples of compact/undifferentiated and spread/differentiated miPS cell colonies. (**b**) The number of undifferentiated (black) and differentiated (white) colonies. All colonies were counted in the dishes. Values are the means ± SD, n = 3. N.S., no significant difference, p > 0.05. **, significant difference, p < 0.01. ***, significant difference, p < 0.001.

The number of undifferentiated miPS cell colonies on 2.5% FA-fixed MEFs was similar to that on MMC-treated MEFs, but there were significantly fewer undifferentiated colonies on ≥5% FA-fixed MEFs (Fig. 4). These data suggest that the biological activity of 2.5% FA-fixed MEFs as nurse cells is similar to that of MMC-treated MEFs.

### Biological activities of miPS

**Figure 5:**
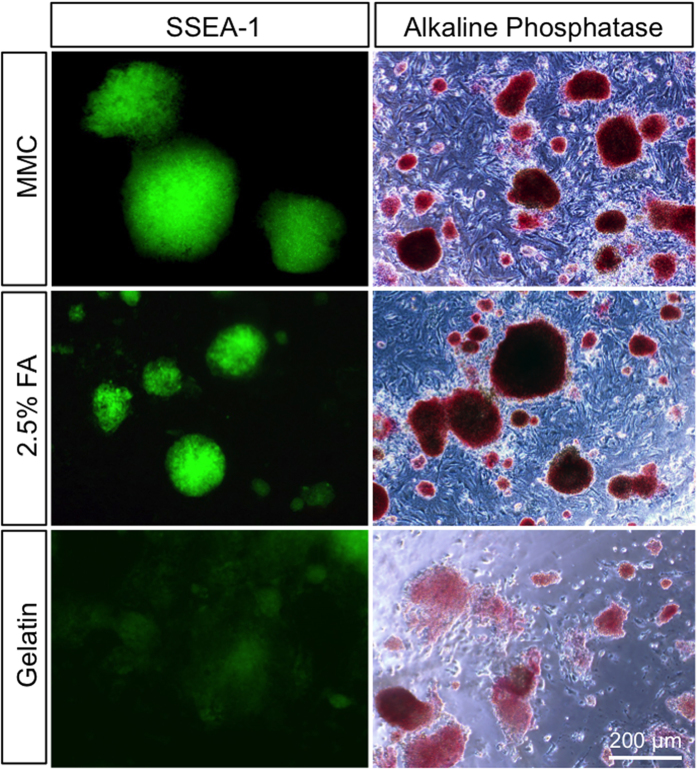
Maintenance of the undifferentiated state of miPS cells on 2.5% FA-fixed MEFs. The expression levels of SSEA-1 and alkaline phosphatase were examined in miPS cells at 3–5 days after seeding on 2.5% FA-fixed MEFs. MMC-treated MEFs and gelatin-coated surfaces were used as control matrices.

**Figure 6:**
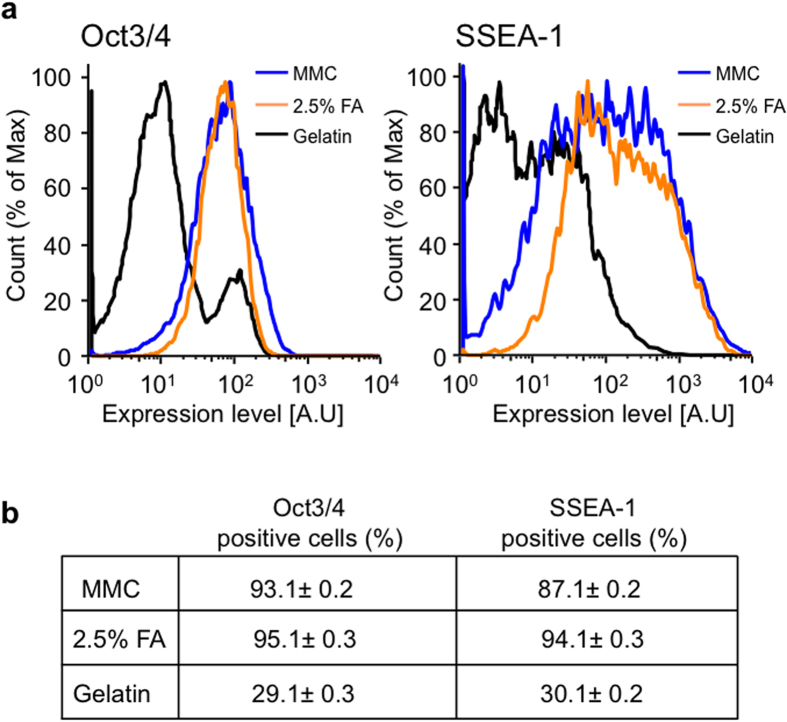
Flow cytometric analysis of miPS cells. (**a**) Flow cytometric analysis of Oct3/4 (left) and SSEA-1 (right) expression in miPS cells cultured on MMC-treated (blue lines) and 2.5% FA-fixed (orange lines) MEFs, and gelatin-coated surfaces (black lines). The analyses were carried out at 3–5 days of culture on the tested matrices. (**b**) The frequency of Oct3/4- and SSEA-1-positive miPS cells. Values are the means ± SD, n = 3.

Next, we further examined the use of 2.5% FA-fixed MEFs to maintain miPS cells in an undifferentiated state. We confirmed sustained expression of alkaline phosphatase, SSEA-1, and other pluripotency markers in miPS cells after 3–5 days of culture on 2.5% FA-fixed MEFs (Fig. 5). Furthermore, flow cytometric analysis showed that ~90% of miPS cells expressed SSEA-1 and Oct3/4 on 2.5% FA-fixed MEFs, whereas only ~30% of miPS cells expressed these markers on gelatin-coated dishes (Fig. 6).

### Long-term culture of miPS cells

**Figure 7:**
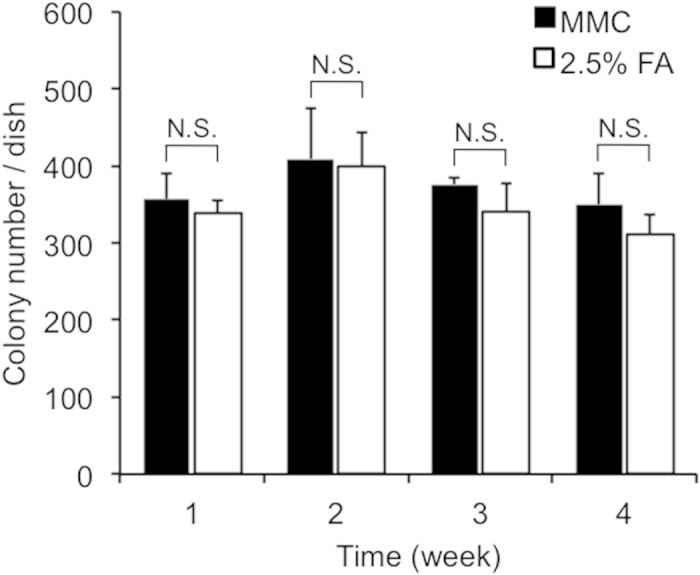
Colony formation of miPS cells on MMC-treated and 2.5% FA-fixed MEFs. All colonies were counted in the dishes. Values are the means ± SD, n = 3. N.S., no significant difference, p < 0.05.

We have already reported that the pluripotency of mouse iPS cells^34^ and human iPS cells^45^ can be maintained on chemically fixed feeder cells over 3 weeks and that the cultured iPS cells are able to form embryonic bodies. Here we cultured mouse iPS cells for more than 4 weeks (cells were passaged every 4 days and, in total, passaged eight times) and quantitatively compared colony formation and expression of a miPS cell pluripotency marker. Figure 7 shows the number of colonies formed on MMC-treated and 2.5% FA-fixed MEFs at different passages. No significant difference was detected for any passage, suggesting the 2.5% FA-fixed MEFs can maintain colony formation of miPS cells even after eight passages.

**Figure 8:**
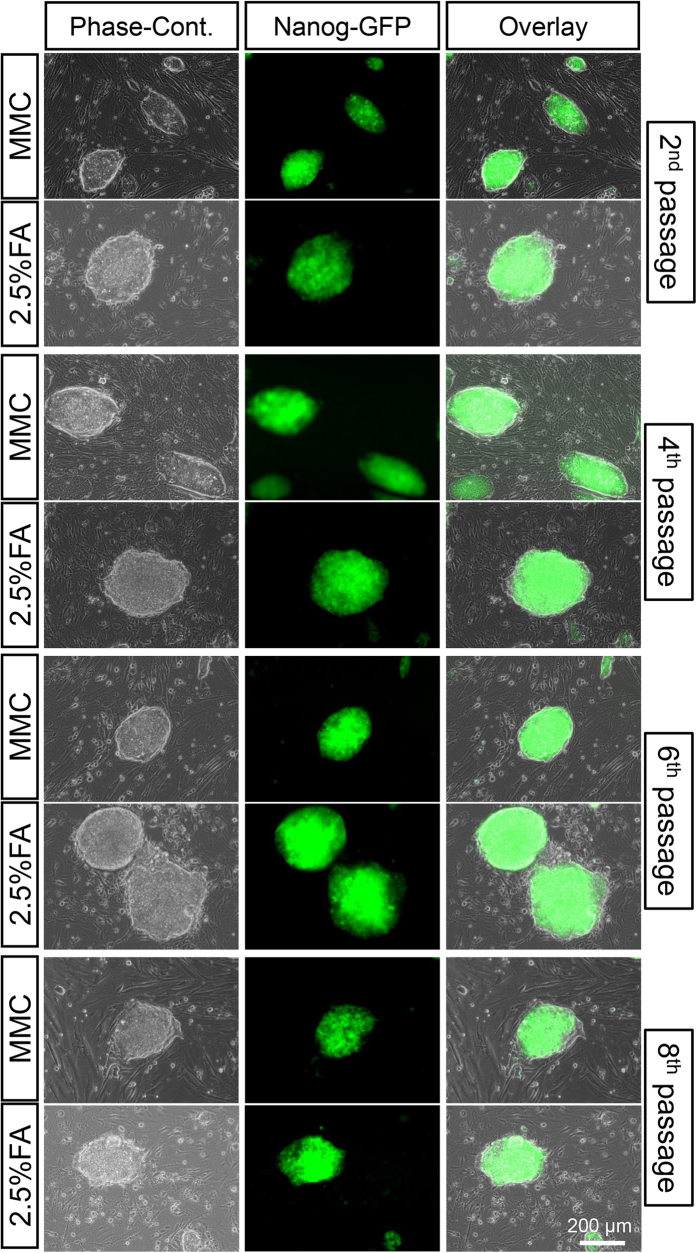
Morphology and Nanog-GFP expression of miPS cells cultured on MMC-treated and 2.5% FA-fixed MEFs at 2nd, 4th, 6th, and 8th passages.

**Figure 9:**
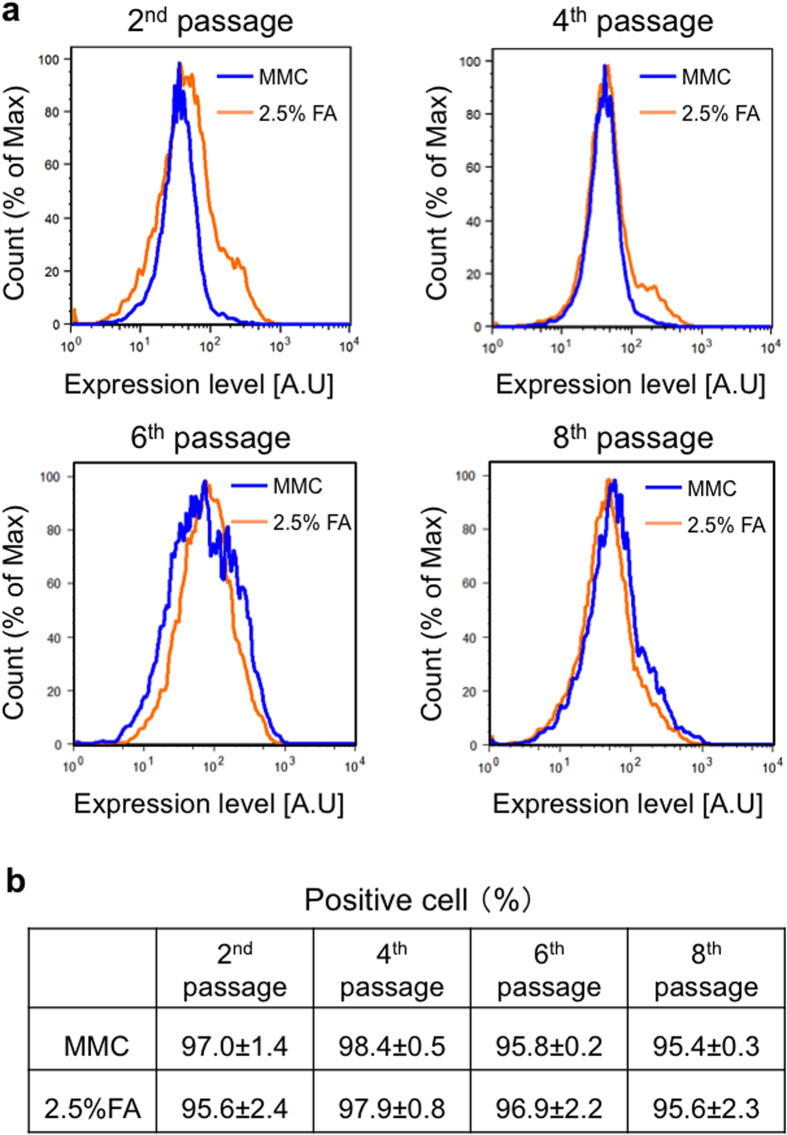
Flow cytometric analysis of miPS. (**a**) Flow cytometric analysis of Oct3/4expression in miPS cells cultured on MMC-treated MEFs (blue lines) and 2.5% FA-fixed MEFs (orange lines) at 2nd, 4th, 6th, and 8th passages. (**b**) The frequency of Oct3/4-positive miPS cells. Values are the means ± SD, n = 3.

Figure 8 shows colony morphology and Nanog expression in miPS cells cultured on MMC-treated and 2.5% FA-fixed MEFs at different passages. Nanog expression was observed in miPS cells at all passages, suggesting that pluripotency of miPS cells can be maintained on chemically fixed feeder cells after long-term cell culture. This result was further confirmed by flow cytometric analysis (Fig. 9). More than 95% of miPS cells cultured on both MMC-treated and 2.5% FA-fixed MEFs were positively stained and no significant difference was detected between them.

Together with the results of the biological assays, these data suggest that 2.5% FA-fixed MEFs can be used as a functional feeder substrate that is equivalent to MMC-treated MEFs.

## Discussion

Tanaka *et al*.^40^ found that membrane molecules move even after chemical fixation. Under common fixation conditions of 4% paraformaldehyde at 25 °C for 30 min, membrane proteins are immobilized easily, although immobilization is dependent on their properties. In addition, antibody-induced clustering of fluorescently labeled membrane molecules, including proteins and lipids, has been observed under fixation conditions. Our results also demonstrated that a low concentration of formaldehyde did not immobilize exogenous proteins.

**Figure 10:**
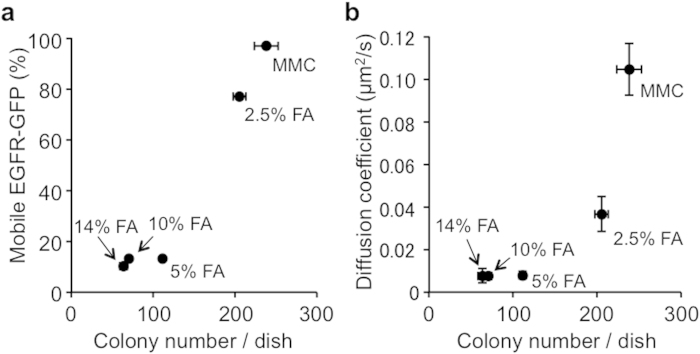
Correlation between the membrane fluidity of MEFs and their biological activity. (**a**) Relationship between the frequency of mobile EGFR-GFP molecules in MEFs and the number of undifferentiated colonies on the corresponding MEFs. (**b**) Relationship between the diffusion coefficient of MEFs and the number of undifferentiated colonies on the corresponding MEFs. The plots were made using the values shown in Figs 2 and 4.

Because we confirmed protein mobility in this study, we hypothesized that the molecular movement of membrane proteins (*i.e*. ligands) is related to feeder cell functions. Consistent with this hypothesis, we found a positive correlation of both the membrane diffusion coefficient and frequency of mobile membrane molecules with the colony formation activity (Fig. 10). Although the critical conditions are unknown for membrane fluidity to promote biological activity, certain diffusivity is required for biological activity.

Decellularized feeders have been employed with human ES and iPS cell culture^46^. Klimanskaya *et al*.^47^ used sodium deoxycholate and Lima *et al*.^48^ used sodium dodecyl sulfate to remove cellular components including DNA. Although they showed that the decellularized extracellular matrix (ECM) could maintain the undifferentiated state, they did not quantitatively compare the activities of MMC-treated and decellularized feeders. Removal of DNA is not considered to be so important for *in vitro* cell culture. Although ECM has the ability to maintain the undifferentiated state of stem cells, because the activity of 2.5% FA-fixed feeder cells is comparable to that of MMC-treated cells, the chemical fixation is useful for *in vitro* stem cell culture.

From a practical viewpoint, our results indicate the importance of the regulation of physical properties for PSC culture substrates. We believe that low concentration FA-fixed MEFs have several advantages for PSC culture as follows. 1) They can be stored as a ready-to-use culture substrate. 2) Because fixed cells are insensitive to proteases, contamination by feeder cells can be prevented, leading to the production of highly pure PSCs. 3) Fixed MEFs provide a low-cost and reproducible PSC culture substrate that is produced by simple procedures. Considering these advantages, our methodology is applicable to large-scale production of PSCs requiring feeder cells.

## Materials and Methods

### Preparation of culture substrates

All animal procedures were conducted according to the Guidelines for the Care and Use of Laboratory Animals of RIKEN and were approved by the RIKEN Institutional Animal Care and Use Committee (Approval ID No.: H23-2-218). To prepare feeder cells as culture substrates, MEFs were established with a standard protocol. Briefly, embryonic day 13.5 mouse embryos were dissected into small pieces, washed twice with phosphate buffered saline (PBS), and then treated with trypsin-EDTA at 37 °C for 30 min with gentle shaking. After removal of aggregated cells by filtration, the cell suspensions were centrifuged to collect the MEFs.

To prepare chemically fixed feeder cells, MEFs were grown to 95% confluence in 60-mm culture dishes, which took about 3 days. After washing with PBS, the cells were fixed with 2.5, 5, 10, or 14% FA diluted in Dulbecco’s modified Eagle’s medium (DMEM) (Wako Pure Chemical Industries, Ltd., Osaka, Japan) at room temperature for 10 min. The fixed MEFs were washed three times with PBS and then incubated overnight in miPS cell culture medium (see below) before use. Conventional feeder cells (MMC-treated MEFs) were prepared as follows. MEFs were grown in 60-mm culture dishes to 80–90% confluence and then treated with 10 μg/mL MMC in DMEM containing 10% fetal bovine serum (FBS) at 37 °C for 3 h. The medium was then replaced with miPS cell culture medium and the cells were incubated overnight at 37 °C before use. To prepare gelatin-coated dishes, a sterile 0.1% gelatin solution was applied to culture dishes, followed by incubation for 40 min at room temperature.

### Culture of miPS cells

The miPS cells were purchased from the RIKEN Cell Bank and cultured on MMC-treated MEFs with miPS cell culture medium (DMEM [Pure Chemical Industries, Ltd., Osaka, Japan] supplemented with 15% [v/v] FBS, 1 mM sodium pyruvate [Invitrogen, Carlsbad, CA, USA], 2 mM glutamine (Invitrogen), 0.1 mM nonessential amino acids [Chemicon, Temecula, CA, USA], and 0.1 mM 2-mercaptoethanol (Sigma, St. Louis, MO, USA), and 1,000 U/mL leukemia inhibitory factor [Chemicon, Temecula, CA, USA]). miPS cell colonies were harvested by gentle pipetting after brief treatment with 0.25% trypsin to avoid detachment of MEFs. Then, the harvested miPS cells were dispersed into small aggregates by pipetting and seeded on 2.5, 5, 10, or 14% FA-fixed MEFs or gelatin-coated dishes.

### Single-molecule imaging

An expression vector for green fluorescent protein-tagged epidermal growth factor receptor (EGFR-GFP), pEGFR-GFP, was constructed as described previously^49^. In this vector, a monomeric mutation was introduced into the GFP-coding region (A206K) to prevent self-association^50^. pEGFR-GFP was transiently transfected into MEFs grown on glass coverslips using Lipofectamine 2000 Reagent (Invitrogen, Carlsbad, CA, USA) according to the manufacturer’s instructions. After incubation overnight, the MEFs were treated with MMC or fixed with 2.5, 5, 10, or 14% FA as described above. Immediately before experimental observations, the solution was replaced with minimum essential medium (Nissui, Tokyo, Japan) containing 1% bovine serum albumin and 5 mM Pipes (pH 7.2).

Single molecules of EGFR-GFP were observed by TIRF microscopy using a Nikon TE2000 inverted fluorescence microscope equipped with a 60 × NA 1.49 objective lens (Plan Apo, Nikon, Tokyo, Japan). The specimens were exposed to a 488-nm wavelength laser, and fluorescence images were acquired using an EM-CCD camera (Image EM; Hamamatsu Photonics, Hamamatsu, Japan) at a temporal resolution of 30.5 ms. All the single-molecule experiments were performed at 25 °C.

### Evaluation of membrane diffusibility and molecular movement

Two-dimensional trajectories of EGFR-GFP molecules in the plane of the basal membrane (Fig. 1a) were reconstructed by custom-made software^51^. This software is often used in single-molecule imaging and functional analysis^52^,^53^,^54^. Mobile and immobile EGFR-GFP molecules were determined as follows. The molecular movement was traced in 200 frames (~6 s), and the molecules that expanded from and stayed within a 200-nm range were regarded as mobile and immobile molecules, respectively (Fig. 1b). The diffusion constant was evaluated as described elsewhere^55^,^56^. Briefly, the mean square displacement (MSD) was plotted from each trajectory against time (*t*). For each molecule, the slope of the first three time points in the MSD *t*plot was used to calculate the diffusion coefficient, *D*, according to the equation MSD_t→0_ = 4*Dt*.

### Detection of pluripotency markers

Nanog expression was directly detected in miPS cells by fluorescence microscopy using a Nanog-GFP reporter construct^57^. Alkaline phosphatase activity was detected using a Vector Red Alkaline Phosphatase Substrate Kit I (Vector Laboratories, Burlingame, CA, USA) after 3.8% FA fixation at room temperature for 10 min, followed by washing with PBS. Immunofluorescence staining of stage-specific embryonic antigen-1 (SSEA-1) was performed with standard procedures using an anti-SSEA-1 antibody (1:200 dilution, clone MC-480; Merck Millipore, Billerica, MA, USA) and Alexa Fluor 546 donkey anti-mouse IgG (H + L) (1:1000 dilution; Invitrogen, Carlsbad, CA, USA) as the secondary antibody.

### Flow cytometric analysis

Flow cytometry was performed to determine the frequency of Oct3/4- and SSEA-1-expressing miPS cells. After 3–5 days of culture on 2.5% FA-fixed MEFs, the miPS cells were harvested by treatment with 0.25% trypsin. To obtain suspensions of single miPS cells, the harvested cells were filtered through a 70-μm cell strainer (Becton Dickinson, Franklin Lakes, NJ, USA). The harvested cells were then stained with anti-Oct3/4 or -SSEA-1 antibodies using a BD Stemflow™ Human and Mouse Pluripotent Stem Cell Analysis Kit (BD Biosciences, San Diego, CA, USA) according to the manufacturer’s protocol. A BD FACSDiva instrument was calibrated before each experiment using Becton Rainbow Calibration Particles (BD Biosciences, San Diego, CA, USA), and the negative gate was set using Alexa Fluor 647 Mouse IgG3 (clone J606), κIsotype Control, and PE Mouse IgM (clone G155-228), κIsotype Control. Before analyzing the stained cells, BD CompBead Plus positive and negative beads were analyzed to facilitate application setup. Non-viable cells were excluded by staining with 0.1% v/v propidium iodide (Sigma-Aldrich, Louis, MO, USA). For long-term cell culture analysis, miPS cells were stained with anti-Oct3/4 antibody after been passaged up to eight times (passaged every 4 days for over 4 weeks).

### Colony formation assay

Approximately 1 × 10^5^ miPS cells (including small aggregates with sizes of less than 50 μm) were cultured on 2.5%, 5%, 10%, or 14% FA-fixed or MMC-treated MEFs in 60-mm culture dishes for 2 days. After fixation with 3.8% FA for 10 min at room temperature, compact and spread colonies (*i.e*., undifferentiated and differentiated colonies, respectively) with sizes exceeding 100 μm were counted under a microscope. For long-term culture of miPS cells on 2.5% FA-fixed and MMC-treated MEFs, compact colony numbers were calculated in 60-mm culture dishes after two, four, six, and eight passages (cells were passaged every 4 days for more than 4 weeks).

## Additional Information

**How to cite this article**: Zhou, Y. *et al*. The significance of membrane fluidity of feeder cell-derived substrates for maintenance of iPS cell stemness. *Sci. Rep*. **5**, 11386; doi: 10.1038/srep11386 (2015).

## Supplementary Material

Supplementary Information

Supplementary Video S1

Supplementary Video S2

Supplementary Video S3

Supplementary Video S4

Supplementary Video S5
