# Supplementary material for: The significance of membrane fluidity of feeder cell-derived substrates for maintenance of iPS cell stemness
Source: Sci Rep. 2015 Jun 12;5:11386. doi: 10.1038/srep11386 (PMC4464345; doi:10.1038/srep11386)
Supplement: Supplementary Information [file srep11386-s1.doc]

Supplementary information

**The significance of membrane fluidity of feeder cell-derived substrates for maintenance of iPS cell stemness**

Yue Zhou^1,2,3+^, Hongli Mao^1+^, Binata Joddar^1^, Nobuhisa Umeki^4^, Yasushi Sako^4^, Ken-Ichi Wada^1^, Chieko Nishioka^5^, Eiki Takahashi^5^, Yi Wang^3^, and Yoshihiro Ito^1,6^*

^1^Nano Medical Engineering Laboratory, RIKEN, 2-1 Hirosawa, Wako, Saitama 351-0198, Japan

^2^School of Nursing, Nanjing University of Chinese Medicine, 138 Xianlin Road, Qixia District, Nanjing, Jiangsu Province 210023, China

^3^Department of Regenerative Medicine, School of Pharmaceutical Science, Jilin University, No.1266 Fujin Road, Changchun 130021, China

^4^Cellular Informatics Laboratory, RIKEN, 2-1 Hirosawa, Wako, Saitama 351-0198, Japan

^5^Support Unit for Animal Experiment, Research Resources Center, RIKEN Brain Science Institute, 2-1 Hirosawa, Wako, Saitama 351-0198, Japan

^6^Emergent Bioengineering Materials Research Team, RIKEN Center for Emergent Matter Science, 2-1Hirosawa, Wako, Saitama 351-0198, Japan

+ equal contribution

Video 1: The movement of EGFR-GFP molecules in 2.5%FA-fixed MEF

Video 2: The movement of EGFR-GFP molecules in 5%FA-fixed MEF

Video 3: The movement of EGFR-GFP molecules in 10%FA-fixed MEF

Video 4: The movement of EGFR-GFP molecules in 14%FA-fixed MEF

Video 5: The movement of EGFR-GFP molecules in MMC-treated MEF
